# Neuromorphological and Neurofunctional Correlates of ADHD and ADD in the Auditory Cortex of Adults

**DOI:** 10.3389/fnins.2022.850529

**Published:** 2022-05-06

**Authors:** Bettina L. Serrallach, Christine Groß, Markus Christiner, Simon Wildermuth, Peter Schneider

**Affiliations:** ^1^Division of Radiology and Nuclear Medicine, Kantonsspital St. Gallen, St. Gallen, Switzerland; ^2^Department of Neuroradiology, University Hospital Heidelberg, Heidelberg, Germany; ^3^Department of Neurology, University Hospital Heidelberg, Heidelberg, Germany; ^4^Jazeps Vitols Latvian Academy of Music, Riga, Latvia; ^5^Center for Systematic Musicology, University of Graz, Graz, Austria

**Keywords:** attention deficit (hyperactivity) disorder, AD(H)D, ADHD subtypes/presentations, magnetic resonance imaging (MRI), magnetoencephalography (MEG), auditory cortex (AC), asynchrony, biomarker

## Abstract

Attention deficit (hyperactivity) disorder (AD(H)D) is one of the most common neurodevelopmental disorders in children with up to 60% probability of prevailing into adulthood. AD(H)D has far-fetching negative impacts on various areas of life. Until today, no observer-independent diagnostic biomarker is available for AD(H)D, however recent research found evidence that AD(H)D is reflected in auditory dysfunctions. Furthermore, the official diagnostic classification systems, being mainly the ICD-10 in Europe and the DSM-5 in the United States, are not entirely consistent. The neuro-auditory profiles of 82 adults (27 ADHD, 30 ADD, 25 controls) were measured *via* structural magnetic resonance imaging (MRI) and magnetoencephalography (MEG) to determine gray matter volumes and activity of auditory subareas [Heschl’s gyrus (HG) and planum temporale (PT)]. All three groups (ADHD, ADD, and controls) revealed distinct neuro-auditory profiles. In the left hemisphere, both ADHD and ADD showed reduced gray matter volumes of the left HG, resulting in diminished left HG/PT ratios. In the right hemisphere, subjects with ADHD were characterized by lower right HG/PT ratios and ADD by a similar right HG/PT ratio compared to controls. Controls and ADD had well-balanced hemispheric response patterns, ADHD a left-right asynchrony. With this study, we present the structural and functional differences in the auditory cortex of adult patients with AD(H)D.

## Introduction

Attention deficit (hyperactivity) disorder (AD(H)D) is one of the most common neurodevelopmental disorders in children and adolescents, with a worldwide prevalence of about 5% (Polanczyk et al., 2007; American Psychiatric Association, 2013). AD(H)D is a chronic and debilitating disorder, affecting all aspects of life and is accompanied by permanent social and emotional overload and high psychological strain (Biederman, 1998; Birnbaum et al., 2005; Biederman and Faraone, 2006; Loe and Feldman, 2007; Wilens et al., 2011). A substantial percentage of up to 60% of children remain affected into adulthood (Weiss and Hechtman, 1993). While hyperactivity may decrease over time, inattention and impulsivity often persist (American Psychiatric Association, 2013).

A wide range of timing deficits have been linked to AD(H)D (Barkley et al., 2001; Smith et al., 2002; McInerney and Kerns, 2003; Falter and Noreika, 2011; Noreika et al., 2013; Lesiuk, 2015), including several timeframes ranging from milliseconds up to years and including auditory, visual, and motor timing as well as temporal foresight problems (Falter and Noreika, 2011; Noreika et al., 2013). Deficits were found in sensorimotor synchronization, duration discrimination, duration reproduction, delay discounting tasks (Noreika et al., 2013), melodic and rhythm processing, and musical performance (Groß et al., 2022). Research has suggested an association between timing deficits and behavioral measures of impulsiveness and inattention indicating that timing deficits may play a key role in AD(H)D (Noreika et al., 2013).

Further AD(H)D-specific features, such as genetic risk variants (Riglin et al., 2016; Demontis et al., 2019), biochemical variations (Elia et al., 2011), neuromorphological (Castellanos et al., 2002; Mostofsky et al., 2002; Nakao et al., 2011; Seither-Preisler et al., 2014; Serrallach et al., 2016; Hoogman et al., 2017, 2019; Firouzabadi et al., 2021; Pereira-Sanchez and Castellanos, 2021) or neurofunctional differences (Kuperman et al., 1996; Seither-Preisler et al., 2014; Serrallach et al., 2016; McVoy et al., 2019; Müller et al., 2019) have been described. This evidence adds to the validity of AD(H)D, which is characterized by the key symptoms of hyperactivity, impulsivity, and/or inattention (American Psychiatric Association, 2013; World Health Organization, 2019), as a neurodevelopmental disorder.

The auditory cortex (AC) is broadly connected and provides detailed information to and receives precise feedback from multiple different brain structures, including attentional networks, demonstrating the interdependence between auditory and attentional functions (Scheich et al., 2011; Seither-Preisler et al., 2014). There is evidence that AD(H)D frequently overlaps with (Central) Auditory Processing Disorder [(C)APD] (Riccio et al., 2005). (C)APD is characterized by difficulties in identifying and discriminating among sounds despite having normal peripheral hearing (Dawes and Bishop, 2009). In addition, patients with (C)APD may show behavioral problems, encompassing inattention and distractibility, features also known from AD(H)D (American Speech-Language-Hearing Association, 2005). Dawes and Bishop (2009) already questioned the reason for this overlap and asked whether auditory processing problems lead to inattention or whether attentional deficits affect auditory perception.

The AC processes auditory information and can be divided into three functional areas, being the core, belt and parabelt regions. The AC follows a hierarchical processing order with the primary information streaming out of the core area to reach the belt region, which in turn connects broadly to the parabelt and auditory-related cortex (Hackett and Kaas, 2004). The Heschl’s gyrus (HG) includes both primary core and secondary belt areas (Seither-Preisler et al., 2014). It is known, that there are large inter-hemispheric and inter-individual morphological variants of HG, including single HG, common stem duplication, complete posterior duplication or multiple duplications (Schneider et al., 2005; Seither-Preisler et al., 2014; Marie et al., 2015; Benner et al., 2017; Turker et al., 2017; Dalboni da Rocha et al., 2020). While the left-hemispheric AC is linked to rapid temporal processing, which is optimal for speech discrimination, the right AC is responsible for spectral processing, which is important for frequency discrimination (Zatorre et al., 2002; Seither-Preisler et al., 2014). The planum temporale (PT) can be found posteriorly to the HG and is part of the auditory association cortex. A decisive role in subserving auditory functions that underlie music and speech processing is attributed to the PT (Meyer et al., 2012). The left PT is seen to be primarily associated with decoding sub-segmental, rapidly changing acoustic cues (about 40 Hz) important for phonemic perception, while the right PT is preferentially responsible for processing supra-segmental, slowly changing cues (about 4 Hz) substantial for prosodic and rhythmic information (Meyer et al., 2012).

The Diagnostic and Statistical Manual of Mental Disorders, Fifth Edition, DSM-5 (American Psychiatric Association, 2013), mainly applied in the United States, distinguishes between ADHD combined presentation, ADHD predominantly inattentive presentation and ADHD predominantly hyperactive-impulsive presentation. In contrast, the International Statistical Classification of Diseases, ICD-10 (World Health Organization, 2019), mainly used in Europe, emphasizes primarily one (sub-)type of this disorder, which is defined by the existence of symptoms of all three behavioral categories (hyperactivity, impulsivity, and attention deficit, F 90.0). However, “attention deficit disorder without hyperactivity” can be found in the ICD-10 under the heading “other specified behavioral and emotional disorders with onset in childhood and adolescence” (F 98.80). This fact reflects the different cultural perception in dealing with the heterogeneity of the disorder (Luo et al., 2019). So far, all AD(H)D presentations/subtypes receive the same therapies mainly consisting of stimulant medication, such as methylphenidate and/or behavior modification therapy (MTA Cooperative Group, 2004).

Emerging literature points out the fact that AD(H)D has far-fetching, long term implications and impacts on various areas of individual life, the society, the economy and the health care system. Comorbid disorders, such as oppositional defiant disorder, conduct disorder, learning disabilities, anxiety disorder, and depression are frequent in individuals with AD(H)D (Biederman et al., 1991). AD(H)D is seen as a significant risk factor for developing cigarette-, alcohol- or drug-use disorders (Wilens et al., 2011). Links between AD(H)D and poor academic and educational outcomes as well as lower socioeconomic status (Biederman and Faraone, 2006; Loe and Feldman, 2007), substantial declines in full-time employment and household income (Biederman and Faraone, 2006) have been reported. A remarkable body of literature has demonstrated that several aspects of AD(H)D such as treatment, increased rates of comorbid psychiatric disorders, high accident rates, work loss, and criminality lead to significant higher direct and indirect medical costs (Birnbaum et al., 2005). Particularly, overdiagnosis systematically leads to inflated healthcare costs due to unnecessary labeling, unneeded tests and inappropriate therapies (Moynihan, 2012). At present, AD(H)D is primarily diagnosed on the basis of patterns of observable behavior, clinical symptoms and diagnostic schemes according to established diagnostic systems (ICD-10 and DSM-5) that not necessarily reflect the underlying neurobiological systems and pathomechanisms (Thome et al., 2012). Hence, it can be expected that a group of disorders with similar symptomatology as AD(H)D but differing pathogenesis are subsumed under the term AD(H)D (Thome et al., 2012). Moreover, with the current diagnostic approach the inter-rater agreement of AD(H)D is low to moderate, a finding that is found across a lot of procedures of psychopathology (Willcutt et al., 2012).

In the light of the above, in 2012, the World Federation of Societies of Biological Psychiatry (WFSBP) task force on biological markers and the World Federation of ADHD called for validated biomarkers of AD(H)D (Thome et al., 2012). The Biomarker Definitions Working Group defines a biological marker (biomarker) as “a characteristic that is objectively measured and evaluated as an indicator of normal biological processes, pathogenic processes, or pharmacologic responses to a therapeutic intervention” (Biomarkers Definitions Working, 2001). As of yet, however, no commonly accepted brain-based method exists that could contribute to a more objective diagnostic procedure for AD(H)D. In last decades, there have been growing attempts in searching for brain-based correlates in neurological and psychiatric diseases in general (Bremner et al., 1995; Müller et al., 2019) and in AD(H)D in particular (Castellanos et al., 2002; Thome et al., 2012; Firouzabadi et al., 2021). There is a large body of literature on differences in neurophysiology including attention, memory, executive functions, language skills, spatial abilities and olfactory functions, risk genes identification, biochemical alterations, proteomic variations and neuroimaging, including structural (conventional, volumetric, and diffusion tensor imaging) and functional (task-based and resting state) magnetic resonance imaging (MRI) (Thome et al., 2012; Firouzabadi et al., 2021; Pereira-Sanchez and Castellanos, 2021). A recent large-scale study using the ENIGMA- (Enhanced Neuroimaging Genetics Through Meta-Analysis) ADHD sample compared the cortical thickness and surface area between 2,246 subjects with ADHD and 1,932 control subjects and found subtle lower surface area in frontal, temporal, and cingulate regions and thinner cortical thickness in the temporal pole and fusiform gyrus in children. These differences in surface area and cortical thickness were not evident in the adolescent or adult group (Hoogman et al., 2019). Lately, artificial intelligence modeling has been increasingly applied to structural and functional imaging with promising results helping to identify imaging features relevant to the diagnosis of AD(H)D (Sun et al., 2018; Firouzabadi et al., 2021).

In previous investigations, we could show that children with AD(H)D, compared with non-affected subjects, show differing morphology of the HG and PT, with decreased gray matter volume of HG and enlarged gray matter volume of the PT resulting in a considerably lower HG/PT ratio. In addition, the primary auditory-evoked responses in the magnetoencephalography demonstrated a characteristic pattern for children with AD(H)D. Compared with non-disorderd peers, children with AD(H)D showed bilateral asynchrony of the P1 evoked response (Seither-Preisler et al., 2014; Serrallach et al., 2016).

Hence, the aim of the present study was to find further evidence and evaluate if the neural correlates of ADHD and ADD in the AC of children can also be found in the AC of adults.

## Materials and Methods

### Subjects and Procedures

Following approval by the responsible ethical committee, participants were recruited by psychiatrists of the Swiss Society for ADHD and by advertisements in relevant ADHD self-help groups in Germany and Switzerland. The inclusion criteria for this study were (1) diagnosed AD(H)D, evaluated by a psychiatrist according to the International Statistical Classification of Diseases and Related Health Problems, 10th Revision, German Modification (ICD-10-GM) and (2) adults ≥ 18 years. Exclusion criteria included: (1) adults with a known neurological disorder, such as epilepsy; (2) pregnant women; (3) metal or metallic implants (e.g., pacemaker) in or around the body; (4) inability to perform a magnetoencephalography (MEG) measurement due to ferromagnetic materials such as not removable retainers or braces, (5) heavy motion artifacts in the MEG or MRI data or (6) claustrophobia.

In 18 cases, in which the written psychiatric diagnosis yielded either no differentiation in subtypes or was unclear regarding subtype classification, the psychiatrist was asked for further specification. In 17 of 18 cases, further specification was obtained according to ICD-10-GM-subtyps [F 90.0 (ADHD) or F 98.80 (ADD)]. In one case the psychiatrist could not provide an unequivocal diagnosis. This patient was excluded from the study.

A comprehensive questionnaire comprising the medical history, medication, therapies, education, profession, drug and addictive drug history assessed the medical and socioeconomic background. The questionnaire was based on our previous study with children/adolescents (Serrallach et al., 2016) and was adapted for adults.

All experimental procedures were in accordance to the Helsinki declaration and were approved by the local ethics committee (EKOS 2018-00002). All participants provided informed consent.

### Magnetic Resonance Imaging

A T1-weighted structural MRI [Siemens, Magnetom Skyra and TrioTim, 3 Tesla, MPRAGE, 176 DICOM slices, sagittal orientation; slice thickness 1 mm, field of view: 256 × 256; matrix size 128 K (16 Bit), repetition time (TR) = 1930 ms, echo time (TE) = 3.47 ms, flip angle 15°] was performed to study the anatomy of the AC. The gray matter surface reconstruction of auditory subareas (HG and PT) was performed using a standardized approach to detect the morphology pattern (Schneider et al., 2002, 2005, 2009; Seither-Preisler et al., 2014; Serrallach et al., 2016). For segmentation, the semi-automatic Brain Voyager software 21.2 (Brain Innovation, B.V, Maastricht, Netherlands) was used. The segmentation procedure and gray matter volume calculation was identical to our previous study (Serrallach et al., 2016). The following steps were conducted: adjustment in contrast and in brightness, accurate correction for inhomogeneity and rotation according to the antero-posterior commissural line. In order to compute group averaged AC surfaces, normalization in Talairach space (Talairach and Tournoux, 1988) was carried out. The superior temporal gyrus (STG), including HG, the anterior superior temporal cortex and the PT were segmented in the sagittal MRI slices along the Sylvian fissure using the standard definition of the landmarks of the auditory cortices and in accordance with established criteria (Schneider et al., 2005, 2009; Seither-Preisler et al., 2014; Wengenroth et al., 2014; Serrallach et al., 2016; Benner et al., 2017). The first complete Heschl’s sulcus (cHS) [large mediolateral extent (> 97%) and pronounced depth] was used as the posterior boundary of HG, and the crescent-shaped first transverse sulcus (FTS) was used as the anterior boundary of HG, thereby dividing the AC into two parts: an anterior auditory area including HG, possibly consisting of several connected HG duplications, e.g., common stem duplication, and aSTG and a posterior area including the PT. Here, we considered the complete HG including potential duplications. HG was separated from aSTG by an anterior plane at *y* = 0. The boundary demarcations for HG and PT of each subject were decided by consensus (BLS and PS). The range of the image gray matter values to be included was calculated individually. A box was placed around left and right AC to generate intensity histograms of these areas. The gray value inclusion range, which was used for surface reconstruction and morphometry, was defined individually. One end of the range was obtained by multiplying the value of the gray matter peak by the factor 0.28, to approximate the change from cerebrospinal fluid to gray matter. The other end was the saddle point between the gray and white matter peaks. The gray and white matter voxels embedded in this inclusion range were marked and used for 3D reconstruction and gray matter voxels for morphometric analysis. In addition to the original parameters gray matter volumes of auditory subareas (right and left HG, right and left PT), and the HG/PT ratios were considered.

### Magnetoencephalography

The response of AC to acoustic stimuli was measured with a Neuromag-122 whole-head MEG system. Before seating the subjects under the dewar helmet of the MEG system, the locations of four head position coils together with a set of 35 surface points including nasion and two pre-auricular points were digitized in a preparation room. Before starting the MEG recordings, the head position inside the dewar helmet was identified. Stimuli were presented binaurally through foam ear pieces (Etymotic ER3). These were connected *via* 90 cm plastic tubes (diameter 3 mm) to small shielded transducers that were fixed in boxes next to the subject’s chair. Eleven representative harmonic complex tones were presented in pseudo-randomized order. Stimuli had a duration of 500 ms and a pseudo-randomized interstimulus interval between 400 and 500 ms. While recording [bandpass filter of 0.00 (DC)–330 Hz; sampling rate of 1,000 Hz], the sounds were presented with a high repetition rate (each sound about 100 times), in order to obtain a sufficient signal-to-noise-ratio. During the measurement (about 25 min) participants were instructed to listen passively to the presented sounds. In order to reduce potential motion artifacts, they were allowed to watch a silent movie. With the variation of the mentioned interstimulus intervals, we aimed to rule out the possibility of crossmodal attention in the AC to visual stimuli, as observed in different frequency bands of brain activity (Luo and Poeppel, 2012), and to avoid the interaction with potential oscillations of brain activity in the computed source waveforms. MEG analysis followed the same procedure as in the previous study of the authors (Serrallach et al., 2016). For data analysis, the Brain Electromagnetic Source Analysis software (BESA Research Software GmbH, Version 7.0; Gräfelfing) was used. The BESA Research Event-Related Fields (ERF) module was used to exclude external artifacts. By applying the automatic Artifact Scan tool, on average about 3–7 noisy/bad channels, and about 10% of all epochs exceeding a gradient of 600 fT/cm × s and amplitudes either exceeding 3,000 fT/cm or falling below 100 fT/cm were rejected from further analysis. Thus, eye blinks, eye movements, cardiac activity, face movements, and muscle tensions as the major part of endogenous artifacts could be accounted for. In addition, a baseline-amplitude, which was calculated over the 100 ms-interval before the onset of the tones, was subtracted from the data. The responses of each subject were combined into a grand average (1,100 artifact-free epochs) with a 100 ms pre-stimulus interval defining the baseline and a time window of 400 ms after stimulus onset. Using a spherical head model (Hämäläinen and Sarvas, 1987; Sarvas, 1987), spatio-temporal source modeling was performed for the P1 response complex (peaking around 60–100 ms after tone onset and existing in both children and adults) by applying one regional source in each hemisphere. Individual adjustment of the fitting interval took place by using the lower and upper half-side lobe around the P1 peak complex and setting the dipole orientation to its maximum. The linear source showing the maximal amplitude was orientated toward the vertex and used for further analyses of P1 latency. Independent of the exact source location in the AC, P1 peak latency show high temporal accuracy (Wengenroth et al., 2014). In addition to the original parameters (right and left P1 latency), an indirect measure of functional lateralization, the absolute P1 latency asynchrony [P1(Peak) (| right – left|)], was considered.

### Statistical Analyses

The statistical analysis is divided into two main sections. First, we provide the descriptive statistics followed by a MANOVA, with which we wanted to reveal whether the variables right HG, left HG, right PT, left PT, right HG/PT, left HG/PT, right P1 latency, left P1 latency and absolute P1 latency asynchrony differ in their mean values in the disorder and control groups. As a follow-up analysis, we performed separate ANOVAs and a discriminant analysis to illustrate how well our variables predict the group membership of our participants. As there were slightly unequal group sizes, we ran Welch-ANOVAs followed by Games-Howell *post hoc* analyses for pairwise group comparisons. Statistical analyses were performed using the software package IBM SPSS Statistics Version 27.0.

## Results

### Subjects

Eighty-two adult subjects fulfilled the inclusion criteria. Twenty-seven subjects were diagnosed with ADHD (17 females; 10 males; mean ± SD age: 42.59 ± 10.03), thirty with ADD (17 females; 13 males; mean ± SD age: 42.33 ± 12.06), and twenty-five were unaffected controls (14 females; 11 males; mean ± SD age: 35.6 ± 6.86).

Compared to controls, the ADHD, and ADD group featured more subjects with (comorbid) mental disease (ADHD: 77.8%; ADD: 73.3%; controls: 4.0%) and more part-time working individuals (ADHD: 80.0%; ADD: 52.6%, and controls: 40.0%), which is consistent with the literature (Biederman et al., 1991, 2006; Biederman and Faraone, 2006). Smoking and illegal drug consumption was more often found in the ADHD group as compared with the ADD, and the control groups, respectively (ADHD: smoking: 40.7%, drug consumption: 11.1%; ADD: smoking: 13.3%, drug consumption: 6.7%, and controls: smoking: 12.0%, drug consumption: 4.0%). The detailed description of the subjects can be found in Table 1. Though, in the general population the male-to-female ratio of AD(H)D is approximately 3:1 (American Psychiatric Association, 2013), more females than males with AD(H)D were willing to participate in this study. Mean values over all groups for the MRI and MEG variables are provided in Table 2.

**TABLE 1:** Description of participants.

Parameters	Categories	ADHD	ADD	Control
N (total/MRI/MEG)		27/27/27	30/30/30	25/25/25
Age	Mean ± SD	42.59 ± 10.03	42.33 ± 12.06	35.6 ± 6.86
Sex	Female	17 (63.0%)	17 (56.7%)	14 (56.0%)
	Male	10 (37.0%)	13 (43.3%)	11 (44.0%)
Handedness	Right	24 (88.9%)	24 (80.0%)	24 (96.0%)
	Left	3 (11.1%)	6 (20.0%)	1 (4.0%)
Mental disease (comorbid)	Yes	21 (77.8%)	22 (73.3%)	1 (4.0%)
	No	6 (22.2%)	7 (23.3%)	24 (96.0%)
	N/a	–	1 (3.4%)	–
Smoking	Yes	11 (40.7%)	4 (13.3%)	3 (12.0%)
	No	16 (59.3%)	26 (86.7%)	22 (88.0%)
Alcohol	Daily/weekly	8 (29.6%)	8 (26.7%)	8 (32.0%)
	Rarely/never	19 (70.4%)	22 (73.3%)	17 (68.0%)
Drug consumption	Yes	3 (11.1%)	2 (6.7%)	1 (4.0%)
	No	23 (85.2%)	28 (93.3%)	24 (96.0%)
	N/a	1 (3.7%)	–	–
Educational level	None/school	1 (3.7%)	2 (6.6%)	1 (4.0%)
	Vocational	18 (66.7%)	17 (56.7%)	9 (36.0%)
	Academic	8 (29.6%)	11 (36.7%)	15 (60.0%)
Employment (if working)	Full-time	3 (20.0%)	9 (47.4%)	15 (60.0%)
	Part-time	12 (80.0%)	10 (52.6%)	10 (40.0%)
Other family members affected as well (ADHD F90.0; ADD F98.80)	Yes	21 (77.8%)	26 (86.7%)	–
	No	6 (22.2%)	4 (13.3%)	
AD(H)D treatment so far	Medication	6 (22.2%)	8 (26.7%)	–
	Psychotherapy	2 (7.4%)	2 (6.7%)	
	Both	19 (70.4%)	19 (63.3%)	
	None	–	1 (3.3%)	
Current AD(H)D medication	Yes	17 (63.0%)	18 (60.0%)	–
	Not currently	10 (37.0%)	12 (40.0%)	

*Group-specific means ± standard deviation (SD) for age and distribution for sex, handedness, comorbid mental diseases, smoking, alcohol, drug consumption, educational level, employment, other affected family members, treatment and current medication.*

**TABLE 2:** Descriptives of the MRT and MEG variables.

Variables	Mean (M)	Standard deviation (SD)
HG (mm^3^) right	4139.77	1118.08
HG (mm^3^) left	3905.26	1037.09
PT (mm^3^) right	2719.57	1086.75
PT (mm^3^) left	3957.91	1166.21
HG/PT ratio right	1.83	1.14
HG/PT ratio left	1.11	0.61
P1 latency (ms) right	58.92	9.7
P1 latency (ms) left	60.66	7.1
Absolute P1 latency asynchrony | R-L| (ms)	5.65	5.24

*Results (mean and standard deviation) for MRI-based gray matter volumes of Heschl’s gyrus (HG), planum temporale (PT), HG/PT ratios in the right and left hemisphere. Results (mean and standard deviation) for MEG-based auditory evoked P1 responses (P1 latency) in the right and left hemisphere and absolute P1 latency asynchrony.*

#### Sex and Groups (ADD, ADHD, and Controls)

Chi-square analysis revealed that there was no association between sex and groups (disorder and control groups) χ^2^(2) = 0.37, *p* = 0.88.

#### Medication and Groups (ADD, ADHD, and Controls)

Chi-square analysis revealed that there was no association between medication and groups (disorder and control groups) χ^2^(2) = 0.88, *p* = 0.70.

### MANOVA

We performed a MANOVA to understand whether the variables right HG, left HG, right PT, left PT, right HG/PT, left HG/PT, right P1 latency, left P1 latency and absolute P1 latency asynchrony differ in their mean values in the disorder and control groups. Using Pillai’s trace there was a significant effect for group membership (control and disorder group) for the right HG, left HG, right PT, left PT, right HG/PT ratio, left HG/PT ratio, right P1 latency, left P1 latency and absolute P1 latency asynchrony *V* = 0.64, *F*(18,144) = 3.78, *p* < 0.001. Since the MANOVA was significant, we ran separate ANOVAs for all nine dependent variables, followed by a discriminant analysis.

### ANOVA

We performed a series of one-way ANOVAs. As there were slightly unequal group sizes, we ran Welch-ANOVAs followed by Games-Howell *post hoc* analyses for pairwise group comparisons in order to illustrate the mean differences of the control and disorder groups. Table 3 illustrates the Welch’s *F*-test and Table 4 represents the Games-Howell *post hoc* analysis.

**TABLE 3:** Welch’s *F*-test ANOVA MRT and MEG variables.

Variables	Welch’s F	p	ω
HG (mm^3^) right	(2, 79) = 6.14	0.003*	0.33
HG (mm^3^) left	(2,79) = 4.54	0.014*	0.29
PT (mm^3^) right	(2, 79) = 4.33	0.016*	0.27
PT (mm^3^) left	(2, 79) = 2.01	0.141	–
HG/PT ratio right	(2, 79) = 5.78	0.005*	0.32
HG/PT ratio left	(2, 79) = 5.58	0.005*	0.32
P1 latency (ms) right	(2, 79) = 8.59	< 0.001*	0.4
P1 latency (ms) left	(2, 79) = 1.50	0.23	–
Absolute P1 latency asynchrony | R-L| (ms)	(2, 79) = 9.38	< 0.001*	0.41

Results for MRI-based gray matter volumes of Heschl’s gyrus (HG), planum temporale (PT), HG/PT ratios in the right and left hemisphere. Results for MEG-based auditory evoked P1 responses (P1 latency) and in the right and left hemisphere and absolute P1 latency asynchrony. *Remains significant after Benjamini–Hochberg correction for multiple comparisons (p < 0.05).

**TABLE 4:** Games-Howell *post hoc* analysis on MRT and MEG variables.

Variables	Group	Means (M) ± standard deviation (SD)	Post-hoc comparisons	t	df	p	r
HG (mm^3^) right	ADHD	3566.56 ± 860.54	ADD vs. controls	0.59	79	0.556	–
	ADD	4344.47 ± 1135.02	ADD vs. ADHD	2.78	79	0.007	0.3
	Controls	4513.20 ± 1137.07	ADHD vs. Controls	–3.24	79	0.002	0.34
HG (mm^3^) left	ADHD	3792.96 ± 1065.32	ADD vs. controls	2.93	79	0.004	0.31
	ADD	3602.10 ± 968.63	ADD vs. ADHD	–0.72	79	0.472	–
	Controls	4390.32 ± 944.80	ADHD vs. controls	–2.16	79	0.033	0.24
PT (mm^3^) right	ADHD	3198.81 ± 1276.48	ADD vs. controls	0.42	79	0.672	–
	ADD	2429.73 ± 848.27	ADD vs. ADHD	–2.78	79	0.007	0.3
	Controls	2549.80 ± 978.70	ADHD vs. controls	2.24	79	0.028	0.26
PT (mm^3^) left	ADHD	3966.30 ± 1041.33	ADD vs. controls	–2	79	0.048	0.22
	ADD	4238.00 ± 1194.91	ADD vs. ADHD	0.89	79	0.377	–
	Controls	3612.76 ± 1211.57	ADHD vs. controls	1.11	79	0.272	–
HG/PT ratio right	ADHD	1.26 ± 0.47	ADD vs. controls	0.57	79	0.572	–
	ADD	2.04 ± 1.02	ADD vs. ADHD	2.71	79	0.008	0.29
	Controls	2.20 ± 1.54	ADHD vs. controls	–3.14	79	0.002	0.33
HG/PT ratio left	ADHD	1.01 ± 0.35	ADD vs. controls	3.16	79	0.002	0.33
	ADD	0.93 ± 0.41	ADD vs. ADHD	–0.5	79	0.622	–
	Controls	1.43 ± 0.88	ADHD vs. controls	–2.61	79	0.011	0.28
P1 latency (ms) right	ADHD	53.78 ± 7.28	ADD vs. controls	–1.94	79	0.055	0.21
	ADD	63.57 ± 8.89	ADD vs. ADHD	4.15	79	< 0.001	0.42
	Controls	58.88 ± 10.39	ADHD vs. controls	–2.07	79	0.042	0.23
P1 latency (ms) left	ADHD	59.67 ± 6.37	ADD vs. controls	–1.48	79	0.142	–
	ADD	62.43 ± 5.56	ADD vs. ADHD	1.48	79	0.143	–
	Controls	59.60 ± 9.08	ADHD vs. controls	0.03	79	0.973	–
Absolute P1 latency asynchrony | R-L| (ms)	ADHD	8.63 ± 6.05	ADD vs. controls	–1.74	79	0.087	–
	ADD	5.20 ± 4.84	ADD vs. ADHD	–2.71	79	0.008	0.29
	Controls	2.96 ± 2.62	ADHD vs. controls	4.28	79	< 0.001	0.44

*Results for MRI-based gray matter volumes of Heschl’s gyrus (HG), planum temporale (PT), and HG/PT ratios in the right and left hemisphere. Results for MEG-based auditory evoked P1 responses (P1 latency) in the right and left hemisphere and absolute P1 latency asynchrony.*

#### ADD vs. Controls

**FIGURE 1:**
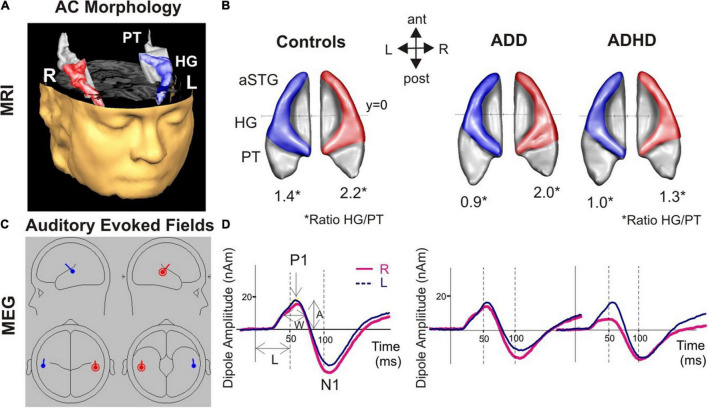
Morphological and functional brain-based correlates for AD(H)D. **(A)** 3D reconstruction of an individual auditory cortex (AC); Heschl’s gyrus (HG), and anterior superior gyrus (aSTG) are colored in blue (left) and red (right), respectively. The planum temporale (PT) and planum polare (anterior to first transverse sulcus) are displayed in gray. **(B)** Top view of group-averaged auditory cortices (L, left; R, right, ant, anterior; post, posterior). The mean ratios of HG/PT gray matter volumes (marked by asterisks “*” indicates the HG/PT ratio) are indicated by numbers. All disorder subgroups (ADHD and ADD) showed downsized left HGs resulting in a diminished left HG/PT. Further, ADHD patients had smaller right HGs and consequently a lower right HG/PT. In contrast, ADD subjects showed no right-hemispheric differences. **(C)** Overview of the MEG dipole localization in the left (blue) and the right hemisphere (red). Group-averaged source waveforms of the P1-N1 complex in response to various sounds for the right (red) and left (blue) hemisphere. **(D)** ADD subjects showed, similar to controls, a well-balanced hemispheric response pattern, ADHD patients a left-right asynchrony with a preceding response in the right hemisphere.

When comparing the MRI-based morphometry, subjects with ADD showed reduced gray matter volumes of the left HG, and enlarged volumes of left PT, consequently resulting in a diminished left HG/PT. The MEG-based auditory evoked magnetic fields of subjects with ADD and controls showed similar time courses. Detailed results are provided in Figure 1 and Table 4.

#### ADD vs. ADHD

Compared to subjects with ADD, subjects with ADHD were characterized by reduced gray matter volumes of the right HG and enlarged volumes of the right PT, resulting in a diminished right HG/PT ratio. Comparing the MEG-based auditory evoked magnetic fields yielded accelerated right P1 latencies in subjects with ADHD compared to subjects with ADD. Further, the absolute P1 latency asynchrony was more pronounced in ADHD group. Detailed results are provided in Figure 1 and Table 4.

#### ADHD vs. Controls

The comparison of the MRI-based morphometry revealed that subjects with ADHD were characterized by a reduced gray matter volume of the right and left HG, and enlarged volumes of right PT, consequently resulting in a diminished right and left HG/PT ratio. The MEG-based auditory evoked magnetic fields showed accelerated right P1 latencies in patients with ADHD leading to a significant absolute P1 latency asynchrony compared to controls. Detailed results are provided in Figure 1 and Table 4.

### Discriminant Analysis

**FIGURE 2:**
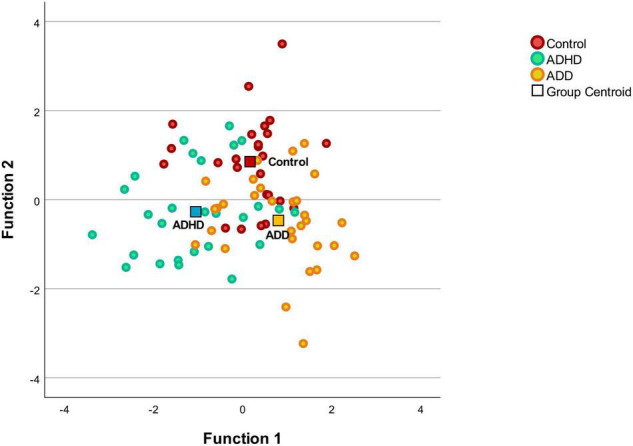
Discriminant plot. Function 1 discriminates the control group and the ADD from the ADHD, while the function 2 discriminates the disorder groups (ADD and ADHD) from the control group. The correlations between the outcomes and the discriminant functions revealed that the loads onto the first function are high for the right P1 latency (*r* = 0.58), the right HG (*r* = 0.43), the right HG/PT (*r* = 0.42), and the right PT (*r* = 0.41). The correlations between the outcomes and the second discriminant functions revealed that the loads onto the second function are high for the left HG/PT (*r* = 0.65), the left HG (*r* = 0.58), and the absolute P1 latency asynchrony (*r* = 0.57).

The MANOVA was followed by a discriminant analysis, which revealed two discriminant functions. The first explained 65.1% of the variance, canonical *R*^2^ = 0.39, whereas the second explained 34.9%, canonical *R*^2^ = 0.25. In combination, these discriminant functions significantly discriminated the groups, Λ = 0.46, χ^2^(*1*8) = 587, *p* < 0.001, and when removing the first function indicated that the second function also significantly differentiated the three groups Λ = 0.75, χ^2^(8) = 21.9, *p* = 0.005. The correlations between the outcomes and the first discriminant functions revealed that the loads onto the first function are high for the right P1 latency (*r* = 0.58), the right HG (*r* = 0.43), the right HG/PT (*r* = 0.42), and the right PT (*r* = 0.41). The correlations between the outcomes and the second discriminant functions revealed that the loads onto the second function are high for the left HG/PT (*r* = 0.65), the left HG (*r* = 0.58), and the absolute P1 latency asynchrony (*r* = 0.57), while lower loads for the left PT (*r* = 0.37), and the left P1 latency (*r* = –0.21) were noted. Since we use a recommended cutoff of 0.40 (Hair et al., 2010) to decide which of the standardized discriminant coefficients are large, the left PT and left P1 latency failed to reach the upper limit. The discriminant plot revealed that the first function separated the control and ADD groups best from the ADHD group, while the second function separated the ADD and ADHD groups from the control group (Figure 2).

## Discussion

In this study, we measured the neuro-auditory profile of 82 adults (27 ADHD, 30 ADD, 25 controls) *via* MRI and MEG. All three groups (ADHD, ADD, and controls) revealed distinct neuro-auditory profiles (Figure 1). In the left hemisphere, both ADHD and ADD showed reduced gray matter volumes of the left HG, resulting in diminished left HG/PT ratios. In the right hemisphere, subjects with ADHD were characterized by lower right HG/PT ratios and ADD by a similar right HG/PT ratios compared to controls. Controls and ADD had a well-balanced hemispheric response pattern, ADHD a left-right asynchrony.

Discriminant analysis revealed that mainly the left HG/PT, the left HG and the absolute P1 latency asynchrony separated the ADD and ADHD groups from the control group, while the right P1 latency, the right HG, the right HG/PT and the right PT separated the control and ADD groups best from the ADHD group (Figure 2). Thus, the structural and functional asymmetry and the lateralization between the right and left hemisphere seem to play an important role in subtype/presentation differentiation. In this study, patients were diagnosed according to the ICD-10-GM. The ICD-10-GM mainly differentiates between two subtypes of AD(H)D (attention deficit hyperactivity disorder and attention deficit disorder without hyperactivity), while the DSM-5 distinguishes between ADHD combined presentation, ADHD predominantly inattentive presentation and ADHD predominantly hyperactive-impulsive presentation. This reflects the still existing uncertainty regarding the exact number of AD(H)D subtypes/presentations. Further, to date, there is no consensus on how to deal with the heterogeneity of AD(H)D. There are studies distinguishing AD(H)D subtypes/presentations (Lee et al., 2008; Seither-Preisler et al., 2014; Serrallach et al., 2016; Groß et al., 2022) and studies considering AD(H)D as a single group (Mostofsky et al., 2002; Nakao et al., 2011; Hoogman et al., 2017, 2019). Differentiating subtypes/presentations in future studies, could help to shed more light on subtype/presentation specific characteristics.

In our preceding study, using the same methodology and similar group sizes, we focused exclusively on children and adolescents, under the age of 17 years (Serrallach et al., 2016). In that study, significant group differences in AC morphology and function were found between the ADHD, the ADD and the control groups, anticipating that auditory functions are indicative for ADHD and ADD (Serrallach et al., 2016). The distinct susceptibility of the AC for ADHD/ADD specific patterns could be explained through the fact that various attentional processes are an integral part within the auditory system. In our current study with adults, all groups (ADHD, ADD, and controls) exhibited a distinct reduction in P1-asynchrony compared to children with ADHD and ADD and unaffected children (Serrallach et al., 2016). It is noticeable, that the reduction is more pronounced in the ADD than the ADHD group.

Combining the results of our preceding study on only children and adolescents (Serrallach et al., 2016) with the current study with only adults, we conclude that characteristic neuro-auditory profiles for ADHD, ADD, and controls can be found in both, the children/adolescent and adult population. We have previously shown that individual differences in the gross morphology of the AC are extremely stable over time and are likely to be mediated by genetic dispositions and/or by prenatal and early environmental influences (Seither-Preisler et al., 2014; Serrallach et al., 2016). The enlarged PTs seen in the patient groups (ADHD and ADD) may originate from diminished or delayed pruning (Iglesias et al., 2005), which potentially leads to oversized anatomical structures and functionally inefficient neural networks (Seither-Preisler et al., 2014; Serrallach et al., 2016; Groß et al., 2022). These morphological anomalies could hinder the build-up of reliable interconnections between bilaterally homotopic regions *via* the corpus callosum (Westerhausen et al., 2009). The development of compensatory, alternative neural connections could result in the observed atypical bilateral asynchronous P1 latencies in ADHD and ADD in children/adolescents (Serrallach et al., 2016), and ADHD in adults.

The currently used behavior-based methodology carries the risk of misdiagnosis with possible negative side effects for individuals, the society, the economy and the health care system. It may have shortcomings in reproducibility and subtype differentiation (Willcutt et al., 2012). As a result of this current diagnostic pathway, AD(H)D tends to be overdiagnosed in boys and underdiagnosed in girls (Bruchmüller et al., 2012; Mowlem et al., 2019). This mentioned overdiagnosis in boys/men and underdiagnosis in girls/women may lead to unnecessary (medication-) treatment in males and lower than necessary access to (medication-) treatment in females (Mowlem et al., 2019). As females often present less externalizing and consequently more internalizing symptoms, they are often misdiagnosed with depression or anxiety (Quinn and Madhoo, 2014). A lack of a clear or a delayed AD(H)D diagnosis and accompanied denied or late treatment such as medication, psychotherapy and psychoeducation may lead to lower educational achievement, long term social problems, higher substance abuse, financial and employment difficulties, higher rates of criminality, imprisonment and motor vehicle accidents (Hamed et al., 2015). This underlines the need for an observer-independent and reliable biomarker (Thome et al., 2012) in the diagnostic work-up of patients with AD(H)D. As revealed by the discriminant analysis, mainly the absolute P1 latency, the left HG/PT and the left HG separated the disorder groups (ADD and ADHD groups) from the control group, while primarily the right side with the right P1 latency, the right HG, the right HG/PT and the right PT separated the control and ADD groups best from the ADHD group (Figure 2). In addition, this combined brain-based morphological and functional approach may lead to an advanced differentiation of AD(H)D subtypes. It has been stated that the development of AD(H)D markers is challenged by etiological and phenotypic complexity and heterogeneity, AD(H)D subtypes, maturation across the age spectrum and comorbidities (Thome et al., 2012; Firouzabadi et al., 2021). Not only do patients with ADHD and ADD differ on a behavioral level (Lahey and Carlson, 1991), but also in auditory processing and music performance (Serrallach et al., 2016; Groß et al., 2022). Compared to controls, children/adolescents with ADHD scored lower in complex rhythmic and melodic perception tasks while children/adolescents with ADD showed no auditory impairments (Serrallach et al., 2016). In rhythmic reproduction, adolescents with ADD performed worse compared to controls, while adolescents with ADHD scored lower in pitch improvisation than controls or adolescents with ADD (Groß et al., 2022). Against this background, and as so far, all AD(H)D presentations/subtypes receive the same therapies, the question arises if more focus should be given in the diagnostic work-up to identify subtypes/presentations in order to develop more presentation/subtype tailored therapies.

We are aware of several limitations in our study. First, our study had a cross-sectional design. Second, the number of subjects in our cohort is rather small. Third, psychiatric diagnoses, even though made by specialized psychiatrists, were performed by different psychiatrists in Germany and Switzerland. Subsequent, longitudinal studies with larger series and homogenized psychiatric diagnoses are needed to confirm these neuromorphological and neurofunctional correlates in the AC. In addition to an independent replication/external validation of the results, future studies should also focus on the question whether the magnitude of the P1 asynchrony is also correlated with the severity of the disorder in adults. Further, as it is known that the morphology of the HG is very variable (Schneider et al., 2005; Seither-Preisler et al., 2014; Marie et al., 2015; Benner et al., 2017; Turker et al., 2017; Dalboni da Rocha et al., 2020) and include single HG, common stem duplication, complete posterior duplication or multiple duplications, future studies should include an assessment of morphotypes and their distribution in specific groups.

In conclusion, until today, no observer-independent diagnostic approach is available for AD(H)D and the official diagnostic classification systems, being mainly the ICD-10 in Europe and the DSM-5 in the United States, are not entirely consistent. We measured the neuro-auditory profile of 82 adults (27 ADHD, 30 ADD, 25 controls) *via* MRI and MEG. We found distinct neuro-auditory profiles for all groups (ADHD, ADD, and controls). In the left hemisphere, both ADHD and ADD showed reduced gray matter volumes of the left HG, resulting in diminished left HG/PT ratios. In the right hemisphere, subjects with ADHD were characterized by lower right HG/PT ratios and ADD by a similar right HG/PT ratio compared to controls. Controls and ADD had well-balanced hemispheric response patterns, ADHD a left-right asynchrony. In the future, observer-independent neuromorphological and/or neurofunctional biomarkers could support the diagnostic work-up of patients with AD(H)D in order to become more definite in diagnosing AD(H)D and its subtypes/presentations.

## Data Availability Statement

The original contributions presented in the study are included in the article, further inquiries can be directed to the corresponding author/s.

## Ethics Statement

The studies involving human participants were reviewed and approved by the Ethikkommission Ostschweiz (EKOS). The patients/participants provided their written informed consent to participate in this study.

## Author Contributions

BS, SW, and PS substantially contributed to the conception and design of the work. BS and PS were involved in the acquisition of the data and performed the neurological analyses. BS, CG, and MC were involved in the interpretation of the findings. MC performed the statistical analysis. BS drafted the work. PS contributed to the figures. MC, CG, PS, and SW performed a critical revision of the manuscript. SW obtained funding. All authors gave their approval to the final version for submission.

## Conflict of Interest

The authors declare that the research was conducted in the absence of any commercial or financial relationships that could be construed as a potential conflict of interest.

## Publisher’s Note

All claims expressed in this article are solely those of the authors and do not necessarily represent those of their affiliated organizations, or those of the publisher, the editors and the reviewers. Any product that may be evaluated in this article, or claim that may be made by its manufacturer, is not guaranteed or endorsed by the publisher.

## References

[B1] American Psychiatric Association (2013). *Diagnostic and statistical manual of mental disorders: DSM-5.* Arlington, VA: American Psychiatric Association.

[B2] American Speech-Language-Hearing Association (2005). *(Central) Auditory Processing Disorders - Technical Report.* Rockville: American Speech-Language-Hearing Association

[B3] Barkley R. A. Edwards G. Laneri M. Fletcher K. Metevia L. (2001). Executive functioning, temporal discounting, and sense of time in adolescents with attention deficit hyperactivity disorder (ADHD) and oppositional defiant disorder (ODD). *J. Abnorm. Child Psychol.* 29 541–556. 10.1023/a:1012233310098 11761287

[B4] Benner J. Wengenroth M. Reinhardt J. Stippich C. Schneider P. Blatow M. (2017). Prevalence and function of Heschl’s gyrus morphotypes in musicians. *Brain Struct. Funct.* 222 3587–3603. 10.1007/s00429-017-1419-x 28397108

[B5] Biederman J. (1998). Attention-deficit/hyperactivity disorder: a life-span perspective. *J. Clin. Psychiatry* 59 (Suppl. 7), 4–16. 9680048

[B6] Biederman J. Faraone S. V. (2006). The effects of attention-deficit/hyperactivity disorder on employment and household income. *MedGenMed* 8:12. PMC1781280 17406154

[B7] Biederman J. Monuteaux M. C. Mick E. Spencer T. Wilens T. E. Silva J. M. (2006). Young adult outcome of attention deficit hyperactivity disorder: a controlled 10-year follow-up study. *Psychol. Med.* 36 167–179. 10.1017/s0033291705006410 16420713

[B8] Biederman J. Newcorn J. Sprich S. (1991). Comorbidity of attention deficit hyperactivity disorder with conduct, depressive, anxiety, and other disorders. *Am. J. Psychiatry* 148 564–577. 10.1176/ajp.148.5.564 2018156

[B9] Biomarkers Definitions Working, G. (2001). Biomarkers and surrogate endpoints: preferred definitions and conceptual framework. *Clin. Pharmacol. Ther.* 69 89–95. 10.1067/mcp.2001.113989 11240971

[B10] Birnbaum H. G. Kessler R. C. Lowe S. W. Secnik K. Greenberg P. E. Leong S. A. (2005). Costs of attention deficit-hyperactivity disorder (ADHD) in the US: excess costs of persons with ADHD and their family members in 2000. *Curr. Med. Res. Opin.* 21 195–206. 10.1185/030079904x20303 15801990

[B11] Bremner J. D. Randall P. Scott T. M. Bronen R. A. Seibyl J. P. Southwick S. M. (1995). MRI-based measurement of hippocampal volume in patients with combat-related posttraumatic stress disorder. *Am. J. Psychiatry* 152 973–981. 10.1176/ajp.152.7.973 7793467 PMC3233767

[B12] Bruchmüller K. Margraf J. Schneider S. (2012). Is ADHD diagnosed in accord with diagnostic criteria? Overdiagnosis and influence of client gender on diagnosis. *J. Consult. Clin. Psychol.* 80 128–138. 10.1037/a0026582 22201328

[B13] Castellanos F. X. Lee P. P. Sharp W. Jeffries N. O. Greenstein D. K. Clasen L. S. (2002). Developmental trajectories of brain volume abnormalities in children and adolescents with attention-deficit/hyperactivity disorder. *Jama* 288 1740–1748. 10.1001/jama.288.14.1740 12365958

[B14] MTA Cooperative Group (2004). National Institute of Mental Health Multimodal Treatment Study of ADHD follow-up: 24-month outcomes of treatment strategies for attention-deficit/hyperactivity disorder. *Pediatrics* 113 754–761. 10.1542/peds.113.4.754 15060224

[B15] Dalboni da Rocha J. L. Schneider P. Benner J. Santoro R. Atanasova T. (2020). TASH: toolbox for the Automated Segmentation of Heschl’s gyrus. *Sci. Rep.* 10:3887. 10.1038/s41598-020-60609-y 32127593 PMC7054571

[B16] Dawes P. Bishop D. (2009). Auditory processing disorder in relation to developmental disorders of language, communication and attention: a review and critique. *Int. J. Lang. Commun. Disord.* 44 440–465. 10.1080/13682820902929073 19925352

[B17] Demontis D. Walters R. K. Martin J. Mattheisen M. Als T. D. Agerbo E. (2019). Discovery of the first genome-wide significant risk loci for attention deficit/hyperactivity disorder. *Nat. Genet.* 51 63–75. 10.1038/s41588-018-0269-7 30478444 PMC6481311

[B18] Elia J. Glessner J. T. Wang K. Takahashi N. Shtir C. J. Hadley D. (2011). Genome-wide copy number variation study associates metabotropic glutamate receptor gene networks with attention deficit hyperactivity disorder. *Nat. Genet.* 44 78–84. 10.1038/ng.1013 22138692 PMC4310555

[B19] Falter C. M. Noreika V. (2011). Interval timing deficits and abnormal cognitive development. *Front. Integr. Neurosci.* 5:26. 10.3389/fnint.2011.00026 21716645 PMC3116141

[B20] Firouzabadi F. D. Ramezanpour S. Firouzabadi M. D. Yousem I. J. Puts N. A. J. Yousem D. M. (2021). Neuroimaging in Attention-Deficit/Hyperactivity Disorder: recent Advances. *AJR Am. J. Roentgenol*. 2021:26316. 10.2214/AJR.21.26316 34406053

[B21] Groß C. Serrallach B. L. Möhler E. Pousson J. E. Schneider P. Christiner M. (2022). Musical Performance in Adolescents with ADHD, ADD and Dyslexia – behavioral and neurophysiological aspects. *Brain Sci.* 12:127. 10.3390/brainsci12020127 35203891 PMC8870592

[B22] Hackett T. A. Kaas J. H. (2004). “The Cognitive Neurosciences III,” in *Auditory Cortex in Primates: Functional Subdivisions and Processing Streams*, 3rd Edn, ed. Gazzaniga M. S. (Cambridge, MA: MIT Press), 1385.

[B23] Hair J. F. Black W. C. Babin B. J. Anderson R. E. (2010). *Multivariate data analysis.* Pearson Education.

[B24] Hämäläinen M. S. Sarvas J. (1987). Feasibility of the homogeneous head model in the interpretation of neuromagnetic fields. *Phys. Med. Biol.* 32 91–97. 10.1088/0031-9155/32/1/014 3823145

[B25] Hamed A. M. Kauer A. J. Stevens H. E. (2015). Why the Diagnosis of Attention Deficit Hyperactivity Disorder Matters. *Front.* P*sychiatry* 6:168–168. 10.3389/fpsyt.2015.00168 26635643 PMC4659921

[B26] Hoogman M. Bralten J. Hibar D. P. Mennes M. Zwiers M. P. Schweren L. S. J. (2017). Subcortical brain volume differences in participants with attention deficit hyperactivity disorder in children and adults: a cross-sectional mega-analysis. *Lancet Psychiatry* 4 310–319. 10.1016/S2215-0366(17)30049-4 28219628 PMC5933934

[B27] Hoogman M. Muetzel R. Guimaraes J. P. Shumskaya E. Mennes M. Zwiers M. P. (2019). Brain Imaging of the Cortex in ADHD: a Coordinated Analysis of Large-Scale Clinical and Population-Based Samples. *Am. J. Psychiatry* 176 531–542. 10.1176/appi.ajp.2019.18091033 31014101 PMC6879185

[B28] Iglesias J. Eriksson J. Grize F. Tomassini M. Villa A. E. (2005). Dynamics of pruning in simulated large-scale spiking neural networks. *Biosystems* 79 11–20. 10.1016/j.biosystems.2004.09.016 15649585

[B29] Kuperman S. Johnson B. Arndt S. Lindgren S. Wolraich M. (1996). Quantitative EEG Differences in a Nonclinical Sample of Children with ADHD and Undifferentiated ADD. *J. Am. Acad. Child Adoles. Psychiatry* 35 1009–1017. 10.1097/00004583-199608000-00011 8755797

[B30] Lahey B. B. Carlson C. L. (1991). Validity of the diagnostic category of attention deficit disorder without hyperactivity: a review of the literature. *J. Learn Disabil.* 24 110–120. 10.1177/002221949102400208 2010673

[B31] Lee S. I. Schachar R. J. Chen S. X. Ornstein T. J. Charach A. Barr C. (2008). Predictive validity of DSM-IV and ICD-10 criteria for ADHD and hyperkinetic disorder. *J. Child Psychol. Psychiatry* 49 70–78. 10.1111/j.1469-7610.2007.01784.x 17979965

[B32] Lesiuk T. (2015). Music perception ability of children with executive function deficits. *Psychol. Music* 43 530–544. 10.1177/0305735614522681

[B33] Loe I. M. Feldman H. M. (2007). Academic and educational outcomes of children with ADHD. *Ambul. Pediatr.* 1 (Suppl.), 82–90. 10.1016/j.ambp.2006.05.005 17261487

[B34] Luo H. Poeppel D. (2012). Cortical oscillations in auditory perception and speech: evidence for two temporal windows in human auditory cortex. *Front. Psychol.* 3:170. 10.3389/fpsyg.2012.00170 22666214 PMC3364513

[B35] Luo Y. Weibman D. Halperin J. M. Li X. (2019). A Review of Heterogeneity in Attention Deficit/Hyperactivity Disorder (ADHD). *Front. Hum. Neurosci.* 13:42. 10.3389/fnhum.2019.00042 30804772 PMC6378275

[B36] Marie D. Jobard G. Crivello F. Perchey G. Petit L. Mellet E. (2015). Descriptive anatomy of Heschl’s gyri in 430 healthy volunteers, including 198 left-handers. *Brain Struct. Funct.* 220 729–743. 10.1007/s00429-013-0680-x 24310352 PMC4341020

[B37] McInerney R. J. Kerns K. A. (2003). Time reproduction in children with ADHD: motivation matters. *Child Neuropsychol.* 9 91–108. 10.1076/chin.9.2.91.14506 12815512

[B38] McVoy M. Lytle S. Fulchiero E. Aebi M. E. Adeleye O. Sajatovic M. (2019). A systematic review of quantitative EEG as a possible biomarker in child psychiatric disorders. *Psychiatry Res.* 279 331–344. 10.1016/j.psychres.2019.07.004 31300243

[B39] Meyer M. Elmer S. Jancke L. (2012). Musical expertise induces neuroplasticity of the planum temporale. *Ann. N. Y. Acad. Sci.* 1252 116–123. 10.1111/j.1749-6632.2012.06450.x 22524348

[B40] Mostofsky S. H. Cooper K. L. Kates W. R. Denckla M. B. Kaufmann W. E. (2002). Smaller prefrontal and premotor volumes in boys with attention-deficit/hyperactivity disorder. *Biol. Psychiatry* 52 785–794. 10.1016/s0006-3223(02)01412-9 12372650

[B41] Mowlem F. D. Rosenqvist M. A. Martin J. Lichtenstein P. Asherson P. Larsson H. (2019). Sex differences in predicting ADHD clinical diagnosis and pharmacological treatment. *Eur. Child Adoles. Psychiatry* 28 481–489. 10.1007/s00787-018-1211-3 30097723 PMC6445815

[B42] Moynihan R. (2012). Too much medicine, not enough mirth. *BMJ* 345:e7116. 10.1136/bmj.e7116 23114064

[B43] Müller A. Vetsch S. Pershin I. Candrian G. Baschera G. M. Kropotov J. D. (2019). EEG/ERP-based biomarker/neuroalgorithms in adults with ADHD: development, reliability, and application in clinical practice. *World J. Biol. Psychiatry* 2019 1–11. 10.1080/15622975.2019.1605198 30990349

[B44] Nakao T. Radua J. Rubia K. Mataix-Cols D. (2011). Gray matter volume abnormalities in ADHD: voxel-based meta-analysis exploring the effects of age and stimulant medication. *Am. J. Psychiatry* 168 1154–1163. 10.1176/appi.ajp.2011.11020281 21865529

[B45] Noreika V. Falter C. M. Rubia K. (2013). Timing deficits in attention-deficit/hyperactivity disorder (ADHD): evidence from neurocognitive and neuroimaging studies. *Neuropsychologia* 51 235–266. 10.1016/j.neuropsychologia.2012.09.036 23022430

[B46] Pereira-Sanchez V. Castellanos F. X. (2021). Neuroimaging in attention-deficit/hyperactivity disorder. *Curr. Opin. Psychiatry* 34 105–111. 10.1097/YCO.0000000000000669 33278156 PMC7879851

[B47] Polanczyk G. de Lima M. S. Horta B. L. Biederman J. Rohde L. A. (2007). The worldwide prevalence of ADHD: a systematic review and metaregression analysis. *Am. J. Psychiatry* 164 942–948. 10.1176/ajp.2007.164.6.942 17541055

[B48] Quinn P. O. Madhoo M. (2014). A review of attention-deficit/hyperactivity disorder in women and girls: uncovering this hidden diagnosis. *Prim. Care Companion. CNS Disord* 16:13r01596. 10.4088/PCC.13r01596 25317366 PMC4195638

[B49] Riccio C. A. Cohen M. J. Garrison T. Smith B. (2005). Auditory processing measures: correlation with neuropsychological measures of attention, memory, and behavior. *Child Neuropsychol.* 11 363–372. 10.1080/09297040490916956 16051564

[B50] Riglin L. Collishaw S. Thapar A. K. Dalsgaard S. Langley K. Smith G. D. (2016). Association of Genetic Risk Variants With Attention-Deficit/Hyperactivity Disorder Trajectories in the General Population. *JAMA Psychiatry* 73 1285–1292. 10.1001/jamapsychiatry.2016.2817 27806167 PMC6485350

[B51] Sarvas J. (1987). Basic mathematical and electromagnetic concepts of the biomagnetic inverse problem. *Phys. Med. Biol.* 32 11–22. 10.1088/0031-9155/32/1/004 3823129

[B52] Scheich H. Brechmann A. Brosch M. Budinger E. Ohl F. W. Selezneva E. (2011). Behavioral semantics of learning and crossmodal processing in auditory cortex: the semantic processor concept. *Hear Res.* 271 3–15. 10.1016/j.heares.2010.10.006 20971178

[B53] Schneider P. Andermann M. Wengenroth M. Goebel R. Flor H. Rupp A. (2009). Reduced volume of Heschl’s gyrus in tinnitus. *Neuroimage* 45 927–939. 10.1016/j.neuroimage.2008.12.045 19168138

[B54] Schneider P. Scherg M. Dosch H. G. Specht H. J. Gutschalk A. Rupp A. (2002). Morphology of Heschl’s gyrus reflects enhanced activation in the auditory cortex of musicians. *Nat. Neurosci.* 5 688–694. 10.1038/nn871 12068300

[B55] Schneider P. Sluming V. Roberts N. Scherg M. Goebel R. Specht H. J. (2005). Structural and functional asymmetry of lateral Heschl’s gyrus reflects pitch perception preference. *Nat. Neurosci.* 8 1241–1247. 10.1038/nn1530 16116442

[B56] Seither-Preisler A. Parncutt R. Schneider P. (2014). Size and synchronization of auditory cortex promotes musical, literacy, and attentional skills in children. *J. Neurosci.* 34 10937–10949. 10.1523/jneurosci.5315-13.2014 25122894 PMC6705250

[B57] Serrallach B. Groß C. Bernhofs V. Engelmann D. Benner J. Gündert N. (2016). Neural Biomarkers for Dyslexia, ADHD, and ADD in the Auditory Cortex of Children. *Front. Neurosci.* 10:324. 10.3389/fnins.2016.00324 27471442 PMC4945653

[B58] Smith A. Taylor E. Rogers J. W. Newman S. Rubia K. (2002). Evidence for a pure time perception deficit in children with ADHD. *J. Child. Psychol. Psychiatry* 43 529–542. 10.1111/1469-7610.00043 12030598

[B59] Sun H. Chen Y. Huang Q. Lui S. Huang X. Shi Y. (2018). Psychoradiologic Utility of MR Imaging for Diagnosis of Attention Deficit Hyperactivity Disorder: a Radiomics Analysis. *Radiology* 287 620–630. 10.1148/radiol.2017170226 29165048

[B60] Talairach J. Tournoux P. (1988). *Co-planar stereotaxic atlas of the human brain : 3-dimensional proportional system: an approach to cerebral imaging.* Stuttgart. New York, NY: Thieme.

[B61] Thome J. Ehlis A. C. Fallgatter A. J. Krauel K. Lange K. W. Riederer P. (2012). Biomarkers for attention-deficit/hyperactivity disorder (ADHD). A consensus report of the WFSBP task force on biological markers and the World Federation of ADHD. *World J. Biol. Psychiatry* 13 379–400. 10.3109/15622975.2012.690535 22834452

[B62] Turker S. Reiterer S. M. Seither-Preisler A. Schneider P. (2017). “When Music Speaks”: Auditory Cortex Morphology as a Neuroanatomical Marker of Language Aptitude and Musicality. *Front. Psychol.* 8:2096. 10.3389/fpsyg.2017.02096 29250017 PMC5717836

[B63] Weiss G. Hechtman L. T. (1993). *Hyperactive children grown up: ADHD in children, adolescents, and adults.* New York, NY: Guilford Press.

[B64] Wengenroth M. Blatow M. Heinecke A. Reinhardt J. Stippich C. Hofmann E. (2014). Increased volume and function of right auditory cortex as a marker for absolute pitch. *Cereb Cortex* 24 1127–1137. 10.1093/cercor/bhs391 23302811

[B65] Westerhausen R. Gruner R. Specht K. Hugdahl K. (2009). Functional relevance of interindividual differences in temporal lobe callosal pathways: a DTI tractography study. *Cereb Cortex* 19 1322–1329. 10.1093/cercor/bhn173 18842665

[B66] Wilens T. E. Martelon M. Joshi G. Bateman C. Fried R. Petty C. (2011). Does ADHD predict substance-use disorders? A 10-year follow-up study of young adults with ADHD. *J. Am. Acad. Child Adolesc. Psychiatry* 50 543–553. 10.1016/j.jaac.2011.01.021 21621138 PMC3104208

[B67] Willcutt E. G. Nigg J. T. Pennington B. F. Solanto M. V. Rohde L. A. Tannock R. (2012). Validity of DSM-IV attention deficit/hyperactivity disorder symptom dimensions and subtypes. *J. Abnorm. Psychol.* 121 991–1010. 10.1037/a0027347 22612200 PMC3622557

[B68] World Health Organization (2019). *“ICD-10 International Statistical Classification of Diseases and Related Health Problems”. 10th revision ed.* Geneva: World Health Organization.

[B69] Zatorre R. J. Belin P. Penhune V. B. (2002). Structure and function of auditory cortex: music and speech. *Trends Cogn. Sci.* 6 37–46. 10.1016/s1364-6613(00)01816-7 11849614

